# Community- and Healthcare-Associated *Clostridium difficile* Infections, Finland, 2008−2013^1^

**DOI:** 10.3201/eid2210.151492

**Published:** 2016-10

**Authors:** Saara M. Kotila, Silja Mentula, Jukka Ollgren, Anni Virolainen-Julkunen, Outi Lyytikäinen

**Affiliations:** National Institute for Health and Welfare, Helsinki, Finland

**Keywords:** Clostridium difficile, bacteria, community-associated infections, healthcare-associated infections, CDIs, incidence, case-fatality rate, population-based study, PCR ribotypes, Finland

## Abstract

We evaluated incidence, case-fatality rate, and trends of community-associated (CA) and healthcare-associated (HA) *Clostridium difficile* infections (CDIs) in Finland during 2008–2013. CDIs were identified in the National Infectious Disease Register, deaths in the National Population Information System, hospitalizations to classify infections as CA or HA in the National Hospital Discharge Register, and genotypes in a reference laboratory. A total of 32,991 CDIs were identified: 10,643 (32.3%) were CA (32.9 cases/100,000 population) and 22,348 (67.7%) HA (69.1/100,000). Overall annual incidence decreased from 118.7/100,000 in 2008 to 92.1/100,000 in 2013, which was caused by reduction in HA-CDI rates (average annual decrease 8.1%; p<0.001). The 30-day case-fatality rate was lower for CA-CDIs than for HA-CDIs (3.2% vs. 13.3%; p<0.001). PCR ribotypes 027 and 001 were more common in HA-CDIs than in CA-CDIs. Although the HA-CDI incidence rate decreased, which was probably caused by increased awareness and improved infection control, the CA-CDI rate increased.

## Abstract 2

Prudent use of antimicrobial drugs in outpatient settings is needed for reducing the burden of infection.

*Clostridium difficile* is a common cause of antimicrobial-associated diarrhea in Finland (*1*) and elsewhere in Europe and Northern America (*2*,*3*). Dissemination of *C. difficile* genotypes with different virulence properties contributes to *C. difficile* infection (CDI) epidemiology (*4*–*6*). PCR ribotype 027 has been associated with more severe CDI outcomes (*7*–*9*), but not all studies have confirmed this finding (*10*). In Finland, hospitalizations associated with CDIs doubled during 1996–2004 (*11*). CDI laboratory-based surveillance was initiated in Finland in 2008, simultaneously with strengthening of infection control according to the European recommendations in several regions (*8*,*12*). During 2008−2010, a 24% reduction was observed in overall CDI incidence in Finland (*13*).

CDI is typically a healthcare-associated (HA) disease, but there are indications that a notable proportion of cases are not associated with recent healthcare exposure (*14*–*16*). Some studies have shown that the incidence and severity of community-associated (CA) CDIs have been increasing (*17*). In Finland, the proportion of CA-CDIs among hospitalized patients in 16 acute-care hospitals was 16% during 2008–2010 (*13*).

The purpose of this study was to compare CA-CDI with HA-CDI in terms of population-based incidence, case-fatality rates, and trends in Finland during 2008−2013. We obtained data from national registers and genotyping results from a reference laboratory.

## Methods

In Finland (population 5.5 million), the national healthcare system is organized into 21 geographically and administratively defined healthcare districts, which have populations ranging from 28,700 to 1.6 million. Sixteen healthcare districts have primary-care and secondary-care hospitals, and 5 provide tertiary-care services.

Since 2008, CDI reporting has been mandatory and all microbiology laboratories in Finland report *C. difficile* findings (positive cultures, toxin production, presence of toxin genes) for stool samples electronically to the National Infectious Disease Register (NIDR) (*8*). Each notification includes specimen date, each person’s unique national identity code, date of birth, sex, and place of residence. In 2008, all laboratories used methods for detecting both TcdA and TcdB and 87% (20/23) used culture of *C. difficile*; 3 laboratories had started to use nucleic acid amplification tests (NAATs) for primary diagnostics (*18*). During 2011–2013, five laboratories were using NAATs as primary diagnostics tests.

The National Hospital Discharge Register is a civil register comprising comprehensive healthcare records provided by all hospitals and primary-care wards in Finland, including outpatient surgery (i.e., day surgery). Each record includes the patient’s national identity code, admission and discharge dates, healthcare provider code, type of service, specialty, and place (home or institution) from which the patient came to the hospital.

Since 2008, clinical microbiology laboratories have been requested to send *C. difficile* isolates from severe cases (CDI-related intensive care, colectomy, or death) (*4*,*12*,*19*) and persistent outbreaks to the national reference laboratory for genotyping. All isolates received by the reference laboratory during 2008–2013 were PCR ribotyped. PCR ribotyping was performed according to the protocol of the Anaerobe Reference Laboratory (Cardiff, UK) (*20*) and by using the Cardiff−European Centre for Disease Prevention and Control (Solna, Sweden) culture collection as reference strains. After gel electrophoresis, band patterns were analyzed by using BioNumerics 3.0–6.6 software (Applied Maths NV, Sint-Martens-Latem, Belgium).

For this study, all notifications of toxin-positive *C. difficile* accompanied by an appropriate national identity code during 2008−2013 were extracted from the NIDR. Using a 3-month time interval, we merged multiple notifications for the same person as a single episode. A total of 32 reports without an appropriate national identity code and 312 reports for persons <1 year of age were excluded. Data from the National Population Information System for 2008–2013 were used as denominators to calculate annual incidence rates and age- and sex-specific average annualized incidence rates, including incidence rate ratios with 95% CIs. Dates of deaths were obtained from the National Population Information System by using the national identity code. Case-fatality rates were calculated by dividing all deaths from any cause <30 days after a positive diagnostic result for CDI was obtained by the total number of CDIs.

We considered as significant values <0.05 without Bonferroni corrections, as per Fisher exact test and χ^2^ test for comparing proportions of PCR ribotypes in CA-CDIs and HA-CDIs. Poisson regression was used to assess whether secular trends in the incidence rates were significant.

On the basis of specimen date for *C. difficile* and national identity code, data for hospitalizations before the *C. difficile*−positive specimen date were obtained from the hospital discharge register. An episode of CDI was classified as HA if the positive specimen was obtained >2 days after admission to a hospital or <4 weeks after discharge and as CA otherwise (obtained outside a hospital, >4 weeks after hospital discharge, or <2 days after admission). Episodes of CDI among residents in long-term care facilities (LTCFs) could be classified as HA only if residents were transferred to a hospital and the positive specimen was obtained <2 days after admission. PCR ribotyping data were linked to NIDR data by using the patient’s date of birth and healthcare district if the date of the specimen was <3 months of the date used for statistics reported to NIDR.

Permission to analyze and link data from the NIDR and the National Hospital Discharge Register was granted by the Ethics Research Committee of the National Institute for Health and Welfare. Because data were already anonymous, informed consent of patients was waived.

## Results

During the 6-year study period, a total of 32,991 incident episodes of CDI (range by year 5,021–6,320) were identified among 29,577 persons. Of the 32,991 CDIs, 10,643 (32.3%) were classified as CA (32.9/100,000 population) and 22,348 (67.7%) as HA (69.1/100,000 population, 3.2/10,000 patient-days).

Of the 10,643 CA-CDIs, 3,166 (29.7%) were among patients whose positive *C. difficile* specimen date was <2 days after admission. Of the 22,348 HA-CDIs, 16,319 (73.0%) were hospital onset (positive specimen date >2 days after hospital admission) and 4,813 (21.5%) were community onset (positive specimen date <4 weeks after hospital discharge). The remaining 1,216 (5.4%) HA-CDIs were in patients transferred from another healthcare institution. For hospital-onset HA-CDIs, median time from hospital admission to positive specimen date was 13 days (range 3−3,785 days), which was similar to that for community-onset HA-CDIs, for which median time from hospital discharge was 13 days (range 1−28 days). Of 4,813 community-onset HA-CDIs, 2,730 (56.7%) were among patients whose positive *C. difficile* specimen date was <2 days after hospital admission.

The average annualized incidence rate for CA-CDIs among persons 15–44 years of age was higher than that for HA-CDIs in the same age group (rate ratio 0.5, 95% CI 0.4–0.7). HA-CDI was most common among persons >45 years of age (Table 1). Overall, the CA-CDI rate for female patients was 1.5 times higher than that for male patients (rate ratio 1.5, 95% CI 1.5–1.6). For persons 15–44 years of age, this difference by sex was ≈2-fold (rate ratio 1.8, 95% CI 1.7–2.0). Although the overall HA-CDI rate was higher for female patients (rate ratio 1.3, 95% CI 1.2–1.3), for persons 45–84 years of age, the rate was higher for male patients.

**Table 1:** Incidence of community-associated and healthcare-associated *Clostridium difficile* infections in patients, by age and sex, Finland, 2008–2013*

Patientage, y	Community-associated								Healthcare-associated						
	Female sex				Male sex				Female sex				Male sex		
	No.	No. person-years	Rate		No.	No. person-years	Rate		No.	No. person-years	Rate		No.	No. person-years	Rate
1–14	252	2,612,610	9.6		248	2,729,947	9.1		126	2,612,610	4.8		152	2,729,947	5.6
15–44	1,551	5,881,383	26.4		886	6,163,161	14.4		578	5,881,383	9.8		533	6,163,161	8.6
45–64	1,655	4,593,089	36.0		1,188	4,554,108	26.1		1,524	4,593,089	33.2		2,047	4,554,108	44.9
65–74	950	1,682,708	56.5		755	1,472,378	51.3		1,917	1,682,708	113.9		2,232	1,472,378	151.6
75–84	1,195	1,176,883	101.5		680	767,551	88.6		4,325	1,176,883	367.5		3,140	767,551	409.1
>84	944	510,899	184.8		339	187,511	180.8		4,224	510,899	826.8		1,550	187,511	826.6
All	6,547	16,457,572	39.8		4,096	15,874,656	25.8		12,694	16,457,572	77.1		9,654	15,874,656	60.8

*Rate is the average annualized incidence rate (episodes/100,000 population).

**Figure 1:**
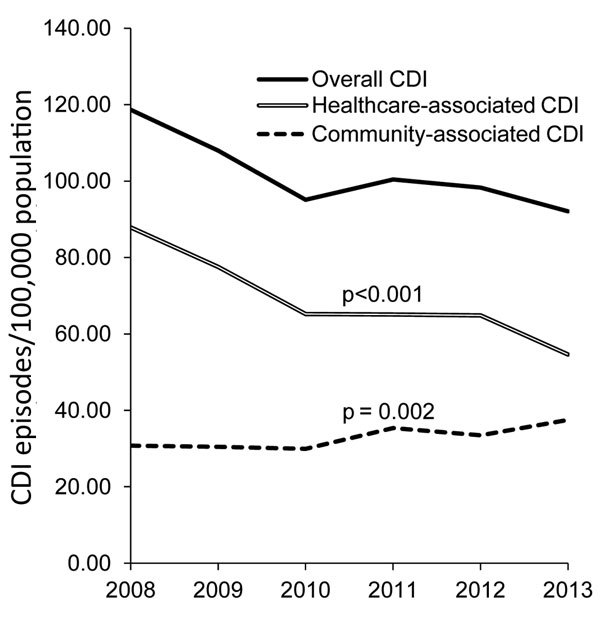
Annual incidence rates of community-associated, healthcare-associated, and overall CDI, Finland, 2008–2013. CDI, *Clostridium difficile* infection.

**Figure 2:**
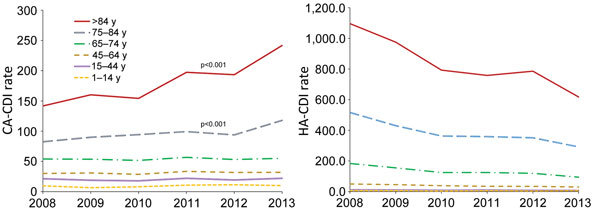
Trends in CDI rates by age group, Finland, 2008–2013. A) CA-CDI; B) HA-CDI. The decrease in the HA-CDI rate was statistically significant (p<0.001) for all age groups except persons 1–14 years of age. CA, community-associated; CDI, *Clostridium difficile* infection; HA, healthcare-associated.

The overall annual incidence rate of CDI decreased significantly from 118.7/100,000 population in 2008 to 92.1/100,000 in 2013 (average annual decrease 4.2%; p<0.01) (Figure 1). The reduction was caused by the decreasing rate of HA-CDI (annual decrease 8.1%; p<0.001). Regionally, the HA-CDI rate decreased for 6 of the 21 healthcare districts and increased in 1 small healthcare district, from 100.3/100,000 population in 2008 to 150.0/100,000 in 2013. The annual incidence rate of CA-CDI increased slightly, from 30.8/100,000 population in 2008 to 37.5/100,000 in 2013 (average annual increase 4.3%; p<0.01). The increase was caused mostly by the increasing trend in persons >74 years of age (Figure 2). The CA-CDI rate increased in 12 healthcare districts, including the healthcare district that showed an increasing HA-CDI trend, and decreased in 1 healthcare district.

Of all CDI episodes during 2008–2013, a total of 3,318 (10.1%) resulted in death within 30 days. The 30-day case-fatality rate was lower for CA-CDIs than for HA-CDIs (3.2% vs. 13.3%; p<0.001), and a difference was observed for all age groups (Table 2). The case-fatality rate for CA-CDI among patients 45–64 years of age was higher for male patients than for female patients, and the case-fatality rate for HA-CDI among patients >64 years of age was higher for male patients than for female patients. The 30-day case-fatality rate was highest for patients with hospital-onset HA-CDIs (15.5%) and lowest for patients with CA-CDIs (2.0%) who had no prior hospitalization since the start of the study in 2008. The 30-day case-fatality rates for CA-CDI and HA-CDI were 3.1% and 14.5%, respectively, in 2008, and 3.7% and 12.7%, respectively, in 2013. The decrease in the case-fatality rate for HA-CDI was significant (p = 0.001), but the case-fatality rate for CA-CDI remained constant.

**Table 2:** Thirty-day case-fatality rates for patients with community-associated and healthcare-associated *Clostridium difficile* infections, by age and sex, Finland, 2008–2013*

Age, y	Community-associated							Healthcare-associated					
	Female sex			Male sex		p value		Female sex			Male sex		p value
	CFR, % (no.)	No. episodes		CFR, % (no.)	No. episodes			CFR, % (no.)	No. episodes		CFR, % (no.)	No. episodes	
1–14	0.4 (1)	252		0.0 (0)	248	1.0		0.8 (1)	126		3.3 (5)	152	0.2
15–44	0.1 (1)	1,551		0.1 (1)	886	1.0		2.1 (12)	578		3.2 (17)	533	0.3
45–64	0.9 (15)	1,655		2.5 (30)	1,188	0.001		7.0 (106)	1,524		8.6 (177)	2,047	0.07
65–74	2.6 (25)	950		4.0 (30)	755	0.1		7.9 (152)	1,917		13.0 (291)	2,232	<0.001
75–84	4.8 (57)	1,195		6.2 (42)	680	0.2		13.3 (574)	4,325		16.5 (518)	3,140	<0.001
>84	10.7 (101)	944		9.7 (33)	339	0.7		17.9 (758)	4,224		23.9 (371)	1,550	<0.001
All	3.1 (200)	6,547		3.3 (136)	4,096	0.5		12.6 (1,603)	12,694		14.3 (1,379)	9,654	<0.001

*Values in parentheses are total no. deaths in category. CFR, case-fatality rate.

During 2008–2013, a total of 16/21 healthcare districts sent 1,523 *C. difficile* isolates for PCR ribotyping. A total of 1,193 *C. difficile* isolates could be linked to CDI episodes in the NIDR data (3.6%), of which 283 were CA and 910 were HA. Among CA and HA isolates, 67 and 99 PCR ribotype patterns were identified, respectively. In both groups, the most frequently identified PCR ribotype was 027. Among the 10 most common PCR ribotypes for the 1,193 isolates (Table 3), PCR ribotypes 027 and 001 were more common among HA isolates, and ribotype 078 was more common among CA isolates. Reasons for requesting typing (severe case or persistent outbreak) were not systematically indicated for isolates, and only 56 were designated to originate from severe cases, of which 43 were HA and 13 were CA. In this subgroup, the most commonly implicated PCR ribotypes were 001 and 027.

**Table 3:** Ten most common PCR ribotypes for *Clostridium difficile* strains with known community or healthcare associations, Finland, 2008–2013

Ribotype	No. (%) strains		
	Community-associated, n = 283	Healthcare-associated, n = 910	All, N = 1,193
027	30 (10.6)	237 (26.0)	267 (22.4)
001	25 (8.8)	154 (16.9)	179 (15.0)
014	19 (6.7)	76 (8.4)	95 (8.0)
023	24 (8.5)	50 (5.5)	74 (6.2)
002	21 (7.4)	42 (4.6)	63 (5.3)
020	20 (7.1)	41 (4.5)	61 (5.1)
078	18 (6.4)	27 (3.0)	45 (3.8)
005	13 (4.6)	25 (2.7)	38 (3.2)
018	12 (4.2)	25 (2.7)	37 (3.1)
011	9 (3.2)	19 (2.1)	28 (2.3)
Other	92 (32.5)	214 (23.5)	306 (25.6)

## Discussion

Our nationwide population-based study aimed to estimate CA-CDI incidence and case-fatality rates for Finland. One third of all CDIs were CA. The overall CDI rate decreased during the study, driven by the decreasing rate of HA-CDI. The CA-CDI rate increased slightly, mostly for elderly persons.

As reported by Lessa et al. in a recent study that assessed CDI burden in the United States (*21*), the comparability of current results with previously published CDI rates is limited by several factors, including differences in CDI definitions and emergence of high-sensitivity NAATs. In our study, an episode of CDI was classified as HA if the positive specimen was obtained <4 weeks after hospital discharge or >2 days after admission and as CA otherwise, in accordance with the European CDI surveillance protocol (*22*). CDIs for which the positive specimen was obtained >4 weeks but <12 weeks after hospital discharge were considered to be CA. If, for better comparability with other population-based CA-CDI studies, the time frame of HA-CDI definition were expanded from 4 weeks to 12 weeks after hospital discharge, the CA-CDI rate would be 24.3 cases/100,000 population (23.8% of all CDIs).

However, our results cannot be compared directly with those of most other studies, which have either separated LTCF residents or combined them with the HA-CDI category. The proportion of CA-CDIs in Finland was lower than in a population-based study that included 10 geographic areas across the United States that participated in the Emerging Infections Program (total population of 11.2 million persons [34.0%] in 2011) (*21*) and in a study in Manitoba, Canada, in 2005–2006 (27%) (*15*). The proportion of CA-CDIs in Finland would have been even lower if we had been able to classify all CDIs of LTCF residents as HA. The CA-CDI rate was at a comparable level in Finland as in Manitoba, where the rate was 23.4 cases/100,000 population. In 10 geographic areas across the United States, the pooled mean crude incidence of CA-CDI was considerably higher (48.2 cases/100,000 population).

In the United States, ≈50% of the 121 laboratories participating in the Emerging Infections Program were using NAATs in 2011 (*21*,*23*). In microbiology laboratories in Finland, the large-scale transition to NAATs took place after the study period in 2014; the proportion of cases diagnosed by using NAATs for CDIs reported to the NIDR increased from <6% in 2013 to 33% in 2014 (*1*). Before use of NAATs, culture and antigen tests were most commonly used in parallel, which indicates that the change in sensitivity has been less drastic than if the antigen tests had been used alone previously. The later transition to NAATs in laboratories in Finland might partially explain the difference in CDI rates compared with those for the United States, but not the difference between proportions of CA-CDIs and HA-CDIs.

Lower levels of antimicrobial drug use could explain to some extent why the CDI rate is lower in Finland (*11*,*24*). It is also likely that awareness of the CDI problem started earlier in the United States than in Finland, which would have influenced diagnostic activity (*11*,*18*). In Finland, <3-fold differences still exist between healthcare districts (unpub. data). Data for the Netherlands and Denmark suggest that current estimations of CA-CDI incidence are largely underestimated because of low diagnostic activity (*25*–*27*).

As reported in previous studies in other countries (*14*,*28*), patients with CA-CDIs in Finland were younger and more likely to be female. In other countries, increased antimicrobial drug use and different use patterns (e.g., treatment of urinary tract infections) have been observed for women (*29*,*30*), which potentially explains the high rate of CA-CDIs in young women. In Finland, the level of fluoroquinolone use has been associated with regional differences in CDI rates; these drugs are used mostly in outpatient care (*31*). Several possible risk factors for CA-CDIs have been suggested, including use of proton pump inhibitors, food contaminated with *C. difficile*, person-to-person and zoonotic transmission, and outpatient healthcare exposure (*32*–*35*). The increase in the CA-CDI rate in Finland was caused mostly by infections in persons >64 years of age, which might represent elderly persons living in the community or in LTCFs. However, the current trend in Finland is to move elderly patients from LTCFs and nursing homes to different types of home care services.

Since 2000, the burden of CDI has increased in North America and in many parts of Europe (*4*,*36*). However, in England and Ontario, Canada, this increasing trend has been overcome by a reduction in HA-CDI rates because of enhanced surveillance and improved control measures (*37**,**38*). We also observed a decrease in the HA-CDI rate in Finland since 2008.

In our study, the 30-day case-fatality rate for CA-CDIs was 3.2%. This rate is higher than the 30-day mortality rate estimated for CA-CDIs in the United States (1.3%) (*21*), most likely because our CA-CDI category included episodes in LTCF residents who probably have several concurrent illnesses and are of an advanced age, but lower than the case-fatality rate (4%) in Örebro, Sweden, earlier during 1999–2000 (*16*). In the study in the United States, CDI was considered to be HA <12 weeks after discharge. In the study in Sweden, patients were followed up for 6–18 months, and case-patients who were not hospitalized in the preceding 60 days were classified as having CA-CDI.

One third of the CA-CDIs in our study were detected in hospitals <2 days after admission. However, we do not know whether CDI was the reason for hospitalization. In Olmsted, Minnesota, USA (*14*) and Connecticut, USA (*28*), hospitalization rates of 40% and 46%, respectively, were observed for CA-CDI patients. However, in both studies the definition of CA-CDI was more exclusive than in our study; this definition considered community-onset case-patients who were not hospitalized in the preceding 3 months (i.e., ≈12 weeks) as having CA-CDIs.

In Finland, the molecular surveillance of CDI aims to support nosocomial outbreak investigations and identify PCR ribotypes that cause severe disease. Thus, only a fraction of isolates are PCR ribotyped. For this study, PCR ribotype was known for only a small, unrepresentative proportion of CDIs, especially with regard to CA-CDI. As in the United States (*21*), hypervirulent PCR ribotype 027 was detected in HA-CDIs and CA-CDIs but was more commonly found in HA-CDIs. PCR ribotype 001 was the second most common ribotype in Finland and was also prevalent in many other countries in Europe during 2008 (*39*). PCR ribotype 078, which has similar genetic properties to hypervirulent type 027 (deletions in the *tcdC* toxin regulator gene), was more common among CA-CDIs than HA-CDIs. This type has been associated with CA-CDI and has severity similar to PCR ribotype 027 in the Netherlands (*6*).

Our study has several limitations. First, there is no national register of all LTCFs in Finland like that for hospitals. Thus, we were not able to classify all LTCF residents with CDIs as HA-CDI; this classification could be made only if residents were transferred to a hospital. This limitation made comparison of the CA-CDI proportion, rate, and case-fatality rate with those of other studies difficult. Second, we did not have data for concurrent conditions and their severity, which would have been needed to evaluate attributable mortality rates, which are known to be more appropriate measurement of CDI outcome. We assessed only 30-day case-fatality rates and compared them with those of previous studies that reported the same measurement. Third, we could ascertain only inpatient healthcare exposure and day surgery, and not whether patients had visited outpatient healthcare facilities. Fourth, it is probable that not all patients with CDIs, especially persons without traditional risk factors, are tested for CDI, which would lead to an underestimation of CA-CDI incidence. Furthermore, the sensitivity of the CDI diagnosis depends largely on the test and algorithm used (*2*). Conversely, it is possible that patients without diarrhea have been tested for CDI, which would result in erroneous inclusion of asymptomatic *C. difficile* carriers as having cases of CDI and overestimation of CDI incidence rate. Moreover, we used the positive specimen date for CDI as a proxy indicator for date of symptom onset, which is also used to determine the origin of CDI cases in the interim CDI case definition for surveillance (*4*). Fifth, the definition used for a CDI episode in our National Infectious Disease Register, which combines multiple reports with a 3-month time interval, might include recurrences and relapses.

One third of CDIs in Finland diagnosed during the 6-year study were CA. Although the HA-CDI rate decreased at the national level, probably in response to improved infection control measures and increased awareness, the CA-CDI rate increased slightly. Prudent use of antimicrobial drugs in outpatient settings, especially for elderly persons, is necessary to reduce the CA-CDI burden, and preventive efforts, such as antimicrobial stewardship campaigns, should also cover long-term care and outpatient settings.
